# Supplementary material for: Predicting visual function by interpreting a neuronal wiring diagram
Source: Nature. 2024 Oct 2;634(8032):113–23. doi: 10.1038/s41586-024-07953-5 (PMC11446822; doi:10.1038/s41586-024-07953-5)
Supplement: Supplementary file 6 — CRF and ERF predictions for individual TmY4 and TmY9 cells. Analogous to Supplementary Data 3, but for TmY target types. Shown are the top four monosynaptic pathways, the strongest pathway passing through each of the top ten intermediary types (ranking from Extended Data Fig. 7), and the trisynaptic pathway Tm1–TmY–Dm3–TmY (see the section entitled Prediction of spatial normalization). [file 41586_2024_7953_MOESM6_ESM.zip › DataS4/TmY9q/720575940611013496.pdf]

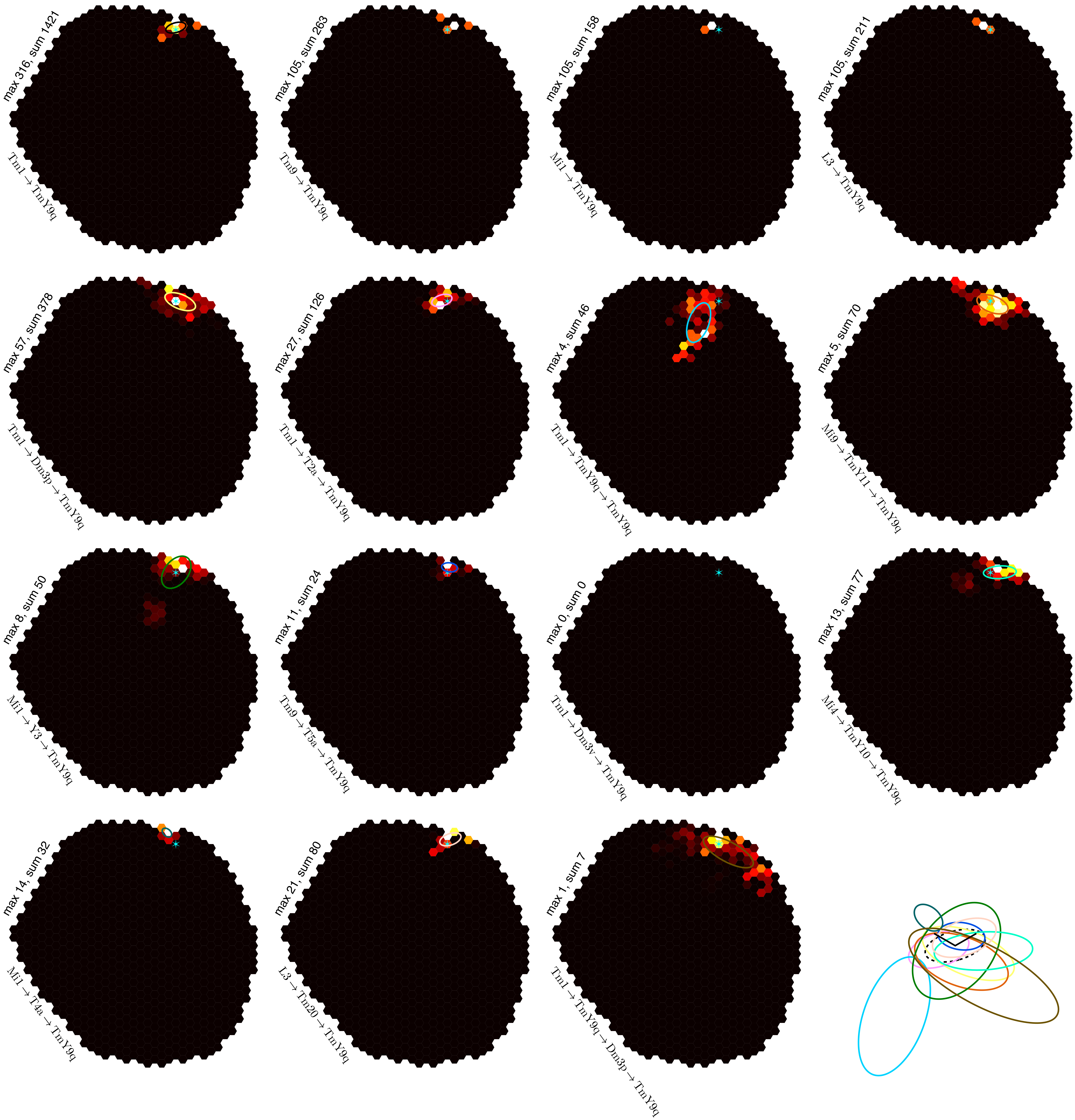

max 316, sum 1421
Tm1 → TmY9q
max 105, sum 263
Tm9 → TmY9q
max 105, sum 158
Mi1 → TmY9q
max 105, sum 211
L3 → TmY9q
max 57, sum 378
Tm1 → Dm3p → TmY9q
max 27, sum 126
Tm1 → T2a → TmY9q
max 4, sum 46
Tm1 → TmY9q → TmY9q
max 5, sum 70
Mi9 → TmY11 → TmY9q
max 8, sum 50
Mi1 → Y3 → TmY9q
max 11, sum 24
Tm9 → T5a → TmY9q
max 0, sum 0
Tm1 → Dm3v → TmY9q
max 13, sum 77
Mi4 → TmY10 → TmY9q
max 14, sum 32
Mi1 → T4a → TmY9q
max 21, sum 80
L3 → Tm20 → TmY9q
max 1, sum 7
Tm1 → TmY9q → Dm3p → TmY9q
